# Enhanced nuclear localization of YAP1‐2 contributes to EGF‐induced EMT in NSCLC

**DOI:** 10.1111/jcmm.17150

**Published:** 2022-01-11

**Authors:** Qiang Guo, Mei‐Yu Quan, Le Xu, Yaxin Cai, Jue‐Ting Cai, Xue Li, Guifeng Feng, Aiping Chen, Weiwei Yang, Qhaweni Dhlamini, Tian‐Fang Jiang, Chengguo Shen, Chengshui Chen, Jin‐San Zhang

**Affiliations:** ^1^ School of Pharmaceutical Sciences Wenzhou Medical University Wenzhou Zhejiang China; ^2^ Key Laboratory of Interventional Pulmonology of Zhejiang Province The First Affiliated Hospital of Wenzhou Medical University Wenzhou Zhejiang China; ^3^ Division of Respiratory Medicine Taizhou Enze Medical Center Enze Hospital Taizhou Zhejiang China; ^4^ Eye Hospital Wenzhou Medical University Wenzhou Zhejiang China

**Keywords:** AKT signalling, EGF, epithelial‐mesenchymal transition, NSCLC, YAP1 isoforms

## Abstract

YAP1, a key mediator of the Hippo pathway, plays an important role in tumorigenesis. Alternative splicing of human YAP1 mRNA results in two major isoforms: YAP1‐1, which contains a single WW domain, and YAP1‐2, which contains two WW domains, respectively. We here investigated the functions and the underlying regulatory mechanisms of the two YAP1 isoforms in the context of EGF‐induced epithelial‐mesenchymal transition (EMT) in non‐small cell lung cancer (NSCLC). Human NSCLC cell lines express both YAP1‐1 and YAP1‐2 isoforms—although when compared to YAP1‐1, YAP1‐2 mRNA levels are higher while its protein expression levels are lower. EGF treatment significantly promoted YAP1 expression as well as EMT process in NSCLCs, whereas EGF‐induced EMT phenotype was significantly alleviated upon YAP1 knockdown. Under normal culture condition, YAP1‐1 stable expression cells exhibited a stronger migration ability than YAP1‐2 expressing cells. However, upon EGF treatment, YAP1‐2 stable cells showed more robust migration than YAP1‐1 expressing cells. The protein stability and nuclear localization of YAP1‐2 were preferentially enhanced with EGF treatment. Moreover, EGF‐induced EMT and YAP1‐2 activity were suppressed by inhibitor of AKT. Our results suggest that YAP1‐2 is the main isoform that is functionally relevant in promoting EGF‐induced EMT and ultimately NSCLC progression.

## 1 INTRODUCTION

Non‐small cell lung cancer (NSCLC) accounts for over 80% of all cases of lung cancer, a leading cause of cancer‐related death worldwide. NSCLC patients frequently harbour activating mutations in the epidermal growth factor receptor (*EGFR*) gene, particularly in the first four exons (exons 18–21) of the EGFR tyrosine kinase domain, which is strikingly high in East Asian populations.^1^ EGFR signalling plays a pivotal role in cellular proliferation, survival and metastatic progression, as well as chemoresistance.^2^

Tumour metastasis and recurrence are main contributors to the high mortality rate of NSCLC,^3^ whereas epithelial‐mesenchymal transition (EMT) is a key initial step of cancer metastasis. EMT refers to a biological process in which epithelial cells lose their polarity and acquire a mesenchymal phenotype and can be induced by intrinsic cell properties as well as extracellular cues, including growth factor stimulation.^4^ During EMT, epithelial cells shift their marker expression profile towards that characteristic of mesenchymal cells, notably decreased E‐cadherin, and increased N‐cadherin as well as vimentin.^3^ In EMT‐associated tumour invasion and metastasis, epithelial‐derived cancer cells lose their epithelial polarity and the ability to adhere to the basolateral membrane and acquire mesenchymal characteristics, such as migration and invasive abilities, allowing them to detach from the primary tumour, enter circulation, and metastasize to distant sites where they may form new lesions.^5^ Therefore, a better understanding of the regulatory mechanisms underlying EMT process will facilitate novel strategies targeting EMT for cancer treatment.

The Hippo pathway is a highly conserved and critical pathway that regulates cell proliferation, apoptosis and self‐renewal.^6^ Numerous studies have established YAP1, the key downstream effector of Hippo pathway, as an important oncogene for tumorigenesis, and a promoter of cancer stemness and metastasis.^7^, ^8^ However, some controversies remain regarding the roles of YAP1 in different cancer cell types and models.^9^ These discrepancies may partly be due to the existence of YAP1 in different isoforms, for which we have recently shown to exhibit distinct functional properties and regulatory mechanisms.^10^

In mammals, at least eight YAP1 protein isoforms derived from alternative mRNA splicing have been identified. YAP1 isoforms can be divided into two subgroups based on the presence of either a single WW motif (YAP1‐1) or two tandem WW domains (YAP1‐2).^11^ The WW domain of YAP1 is responsible for interactions with a number of PPxY motif‐containing proteins in the Hippo pathway (where P is proline, x is any amino acid and Y is tyrosine),^12^ such as LATS1/2,^13^ AMOT,^14^ WBP2^15^ and PTPN14.^16^ The presence of either a single WW or double WW domains may influence the affinity as well as the specificity of YAP1 during its interactions with these PPxY motifs.^17^ We recently demonstrated that, under high cell density, the YAP1‐2 protein exhibits stronger interactions with several negative regulators making it less stable compared to the YAP1‐1. YAP1‐2 protein is preferentially degraded under high cell density, and likely in the setting of dense solid tumour.^10^ Our data suggest that YAP1‐1 and YAP1‐2 isoforms are differentially regulated by the upstream Hippo pathway and may be subject to regulation by diverse stimuli such as cell‐cell contact, mechanical cues, as well as EMT. Although several studies have documented direct and indirect roles of YAP1 in the EMT process,^18^, ^19^ the detailed functional properties and regulatory mechanisms underlying each specific YAP1 isoform during EMT remain unclear.

In our current work, we examined the role of YAP1 in NSCLC with a focus on the YAP1‐1 and YAP1‐2 isoforms in the EGF‐induced EMT process. Our results indicated that YAP1 plays an essential role in maintaining the EMT phenotype such as cell migration and EMT marker expression in NSCLC. However, YAP1‐2 exhibited a stronger effect than YAP1‐1 in promoting EGF‐induced EMT in NSCLC cells. Mechanistically, we showed that activation of AKT pathway by EGF treatment preferentially leads to YAP1‐2 stabilization and its nuclear localization.

## 2 MATERIALS AND METHODS

### 2.1 Plasmids

To knockdown the endogenous YAP1 expression, we used the pLKO.1 lentivirus expression system (Sigma). The YAP1 shRNA targeting sequence (listed in the 5′ to 3′ direction) is CCCAGTTAAATGTTCACCAAT (#1) and GCCACCAAGCTAGATAAAGAA (#2). Full‐length YAP1‐1γ and YAP1‐2γ were generated and subcloned into the lentiviral expression vector pLenti6.3 (Invitrogen), as previously described.^10^

### 2.2 Antibodies and reagents

The primary antibodies and their commercial suppliers are YAP1 (cat. 14074S), p‐YAP1 (s127) (cat. 13008S), E‐cadherin (cat. 3195S), Snail (cat. 3895S), AKT (cat. 4691S), p‐ATK (s473) (cat. 4060S), EGFR (cat. 2646S) and p‐EGFR (Tyr1068) (cat. 3777S) from Cell Signaling Technolog, Vimentin (cat. 10366–1‐AP) from Proteintech (USA), GAPDH (cat. MB001‐100) from Bioworld, Histone H3 (cat. AF0009) from Beyotime (CHN), Goat anti‐Rabbit IgG‐H&L (Alexa Fluor^®^ 488) (cat. ab150077) from Abcam. Recombinant human EGF was purchased from Peprotech (cat. AF‐100–15–100, Peprotech). MK2206 was purchased from Selleck (cat. S1078, Selleck) and DAPI (D1306) from Thermo Fisher.

### 2.3 Cell culture, transfection and treatment

The NSCLC cell lines A549, H460 and H1975 were purchased from American Type Culture Collection (ATCC). The cells were maintained under the recommended culture conditions and transfected as previously described.^20^ For EGF treatment, the cancer cells were treated with 25 ng/ml EGF for 3 days to induce the EMT process. All experiments were performed three times.

### 2.4 Lentiviral packaging, transduction and stable cell selection

Lentiviral packaging, host cell transfection and puromycin selection of stably cells containing pLKO‐ShRNA were performed as previously described.^20^ For the stable reconstituted expression of specific YAP1 isoforms, lentiviral particles carrying pLenti6.3‐Flag‐YAP1 cDNA encoding either YAP1‐1γ or YAP1‐2γ were used to transduce A549‐shYAP1 cells. The cells were then selected in culture medium supplemented with blasticidin (5 μg/ml), and the surviving blasticidin‐resistant cells were used as stable overexpression cells.

### 2.5 RNA isolation, real‐time PCR and YAP1 isoform detection

Total RNA was extracted with RNAiso Plus (TaKaRa, JPN). The PrimeScript RT Reagent Kit (TaKaRa, JPN) was used for cDNA synthesis. Real‐time PCR was carried out using the CFX96 Real‐Time System (Bio‐Rad) and SYBR Premix Ex Taq (TaKaRa, JPN). The gene‐specific primers used in this study are as follows: total YAP1 none discriminative of isoforms (forward: 5’‐CAAATCCCACTCCCGACAG‐3’, reverse: 5’‐GTCAGTGTCCCAGGAGAAAC‐3’); E‐cadherin (forward: 5’‐AATGCCGCCATCGCTTAC‐3’, reverse: 5’‐ ACCAGGGTATACGTAGGGAAACTCT‐3’); Vimentin (forward: 5’‐ GAGGATCTGGAATTCGGATCC‐3’, reverse: 5’‐ ACGCGTCGACTTATTCAAGGT‐3’); Snail (forward: 5’‐ GTTTACCTTCCAGCAGCCCTAC‐3’, reverse: 5’‐ AGCCTTTCCCACTGTCCTCAT‐3’); YAP1‐1 (forward: 5’‐ AGGTTGGGAGATGGCAAAG‐3’, reverse: 5’‐ GATTCTCTGGTTCATGGCTGA‐3’); YAP1‐2 (forward: 5’‐ ACAAGCCATGACTCAGGATG −3’, reverse: 5’‐ TGTTTCACTGGAGCACTCTG‐3’); CTGF (forward: 5’‐CTTCTGTGACTTCGGCTCC‐3’, reverse: 5’‐ACGTGCACTGGTACTTGC‐3’); CYR61 (forward: 5’‐CAAGGAGCTGGGATTCGATG‐3’, reverse: 5’‐AAAGGGTTGTATAGGATGCGAG‐3’); and GAPDH (forward: 5’‐ACATCGCTCAGACACCATG‐3’, reverse: 5’‐TGTAGTTGAGGTCAATGAAGGG‐3’). All the values were normalized to GAPDH.

### 2.6 Immunofluorescence staining and imaging

Cells were grown on glass‐bottom cell culture dishes (NEST, CHN) for immunofluorescence studies. Staining was carried out as previously described with a primary antibody against YAP1 at 1:200, and goat anti‐rabbit Alexa Fluor^®^ 488 as the secondary antibody at 1:1000. DAPI was used for counterstaining. Confocal images were obtained with a Leica SP8 confocal microscope and Suite‐Advanced Fluorescent software.

### 2.7 Scratch healing assay

Cells were seeded and cultured in complete medium in six‐well plates (Corning) until they reached >90% confluence. The cells were scratched with a 20‐μl plastic pipette tip. After thorough and gently washing with PBS, the cells that migrated into the wounded areas were imaged at 0 h, 24 h, 48 h and 72 h, respectively (Leica DMIL LED, Leica DFC3000G). The migrated distance was measured using ImageJ 1.48 v software by comparing the images from 0 h to those from the indicated points.

### 2.8 Trans‐well assay

The cell migration assay was performed using a Boyden chamber in a 24‐well plate designed by Cell Biolabs Inc. according to the manufacturer's instructions. Briefly, for each condition, cells were suspended at 5 × 10^5^ cells per ml in serum‐free RPMI1640 medium and added 200 ml to the upper chamber of each well. The same medium supplemented with 10% serum was added to the lower chamber of each well as a chemoattractant. After 24 h, the cells that migrated to the lower chamber of each well were stained using crystal violet cell staining solution. The stained cells were imaged and counted for statistical analysis.

### 2.9 Statistical analysis

The real‐time PCR, MTT assays and tumour growth curve data are presented as the mean ± SD. *p* values showing differences were calculated by an unpaired two‐tailed *t* test, and those showing no differences were calculated by a one‐tailed *t* test.

## 3 RESULTS

### 3.1 YAP1 expression positively correlates with EGFR activity and NSCLC malignancy

**FIGURE 1:**
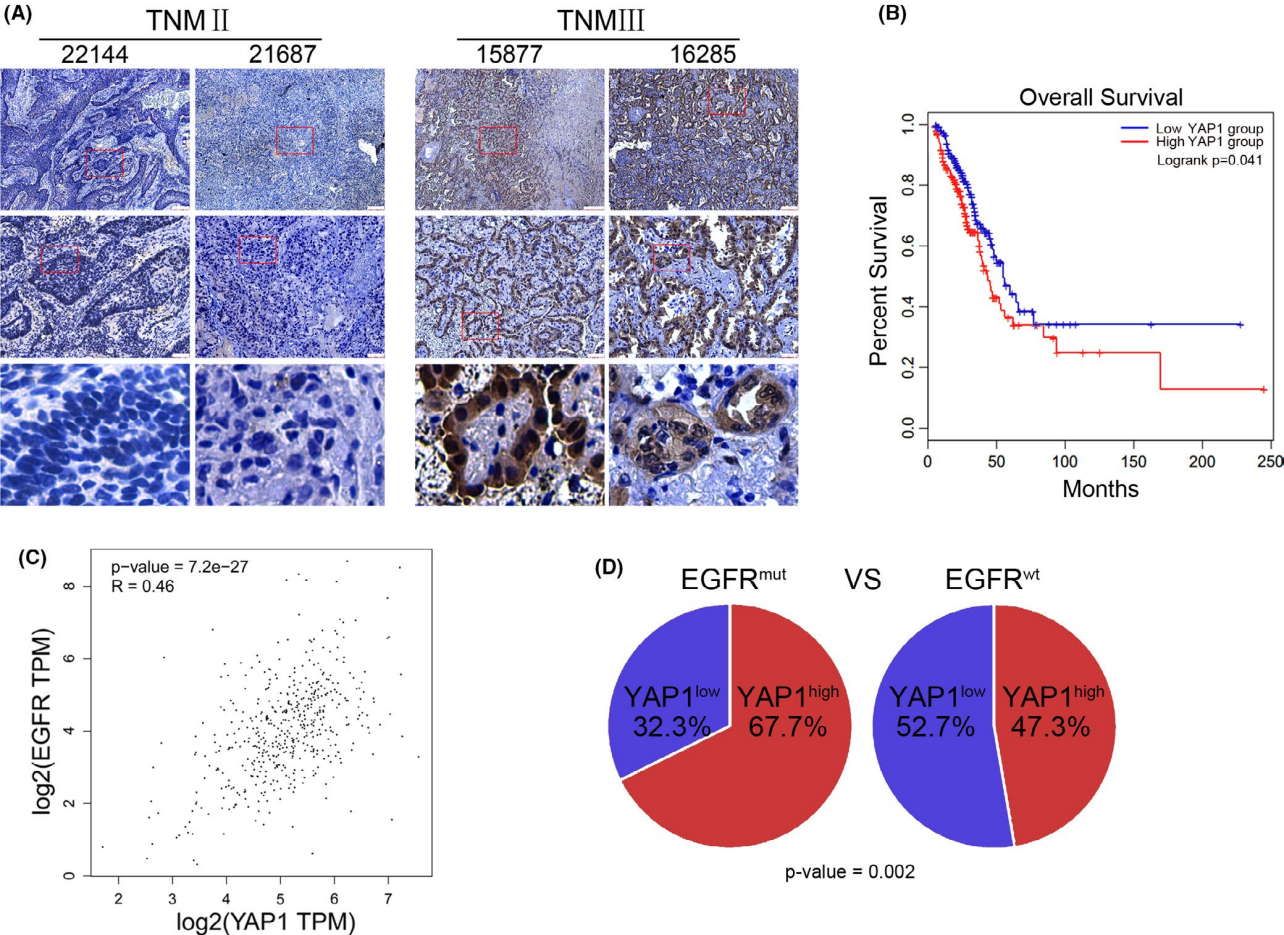
YAP1 expression positively correlates with EGFR Activity and NSCLC Malignancy. (A) The patients' sample sections were subjected to IHC detection with YAP1 antibodies. (B) The overall survival (OS) rates in patients with high YAP1 expression and low YAP1 expression (TCGA database). (C) Correlation analysis of EGFR and YAP1 expression (TCGA database, Correlation Coefficient: spearman, *N* = 533, *R* = 0.46). (D) Expression analysis of YAP1 in EGFR mutated patients vs. wild‐type patients (TCGA database, 44/(21 + 44) vs. 210/(210 + 234), *p* < 0.01)

We collected clinical samples from 4 NSCLC patients to analyse the expression of YAP1 by immunohistochemistry. The results showed that the YAP1 expression in TNM stage 3 patients (15877 and 16285) was higher than that of TNM stage 2 patients (22144 and 21687; (Figure 1A,B). Kaplan‐Meier analysis was used to analyse the relationship between YAP1 expression and patient survival in NSCLC (data from TCGA). We found that the overall survival (OS) in the patients with high YAP1 expression was significantly lower than in those with lower YAP1 expression (Figure 1C) suggesting that high YAP1 expression correlates to poor survival (data from TCGA). In lung adenocarcinoma, oncogenic EGFR expression and mutations co‐occur with many other oncogene alterations. Here, we found that the expression of YAP1 and EGFR is positively correlated (Figure 1C) and patients harbour EGFR mutations are more likely to have higher YAP1 expression, although the TCGA data do not discern YAP1‐1 from YAP1‐2 isoforms (Figure 1D).

### 3.2 EGF upregulates YAP1 expression and promotes EMT in NSCLC

**FIGURE 2:**
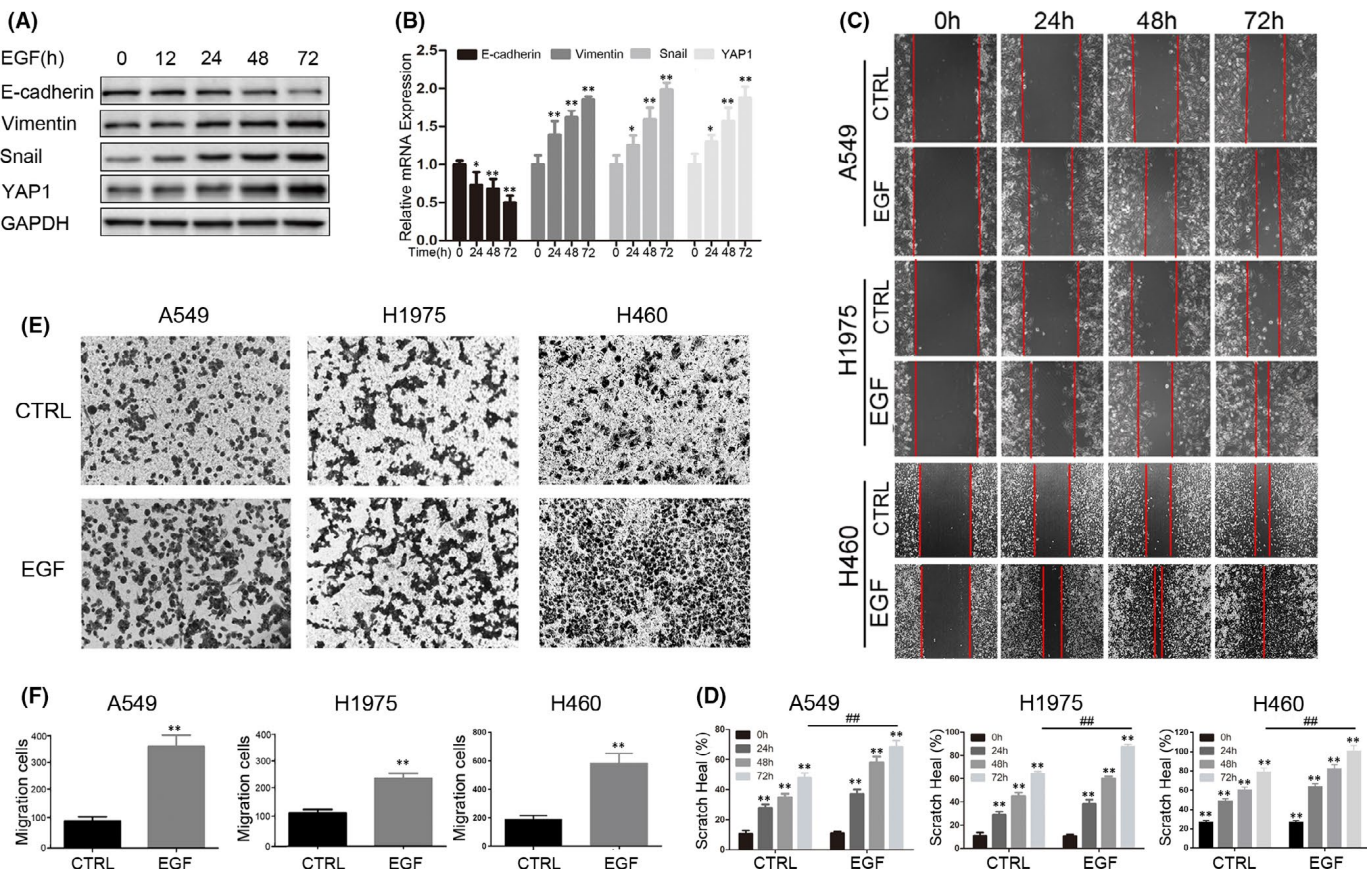
EGF upregulates YAP1 and promotes lung cancer epithelial‐mesenchymal transition (EMT). (A,B) A549 cells were treated with 25 ng/ml EGF according to the time table, Western blots (A) and qPCR (B) were used to detect the expression of EMT‐related proteins. (C,E) A549, H460 and H1975 cells were pre‐treated with 25 ng/ml EGF for 72 h, the scratch healing assay (C) and trans‐well assay (E) were performed to detect the migration ability. (D,F) Statistic analysis of the scratch healing assay (D) and trans‐well assay (F). *p < 0.05, **p < 0.001, and ^##^ *p* < 0.001 compared to the linked group

To determine the effect of EGF on the EMT process in NSCLC, A549 cells were treated with EGF (25 ng/ml) for 0, 12, 24, 48 and 72 h, respectively. Western blots and qPCR analyses showed that both the protein and mRNA levels of YAP1 were upregulated by EGF treatment. The mesenchymal markers Vimentin and Snail were also upregulated, while the epithelial marker E‐cadherin was downregulated (Figure 2A,B). Scratch healing assays (Figure C,D) and trans‐well assays (Figure 2E,F) were then performed using cells pre‐treated with EGF for 72 h to initiate the EMT process. The results showed that the wound healing efficiency as well as the migrated cell number of A549, H460 and H1975 were dramatically increased upon EGF treatment. These results indicated that EGF stimulation induces EMT in NSCLC cells and upregulates YAP1 expression.

### 3.3 YAP1 knockdown impairs EGF‐induced EMT

**FIGURE 3:**
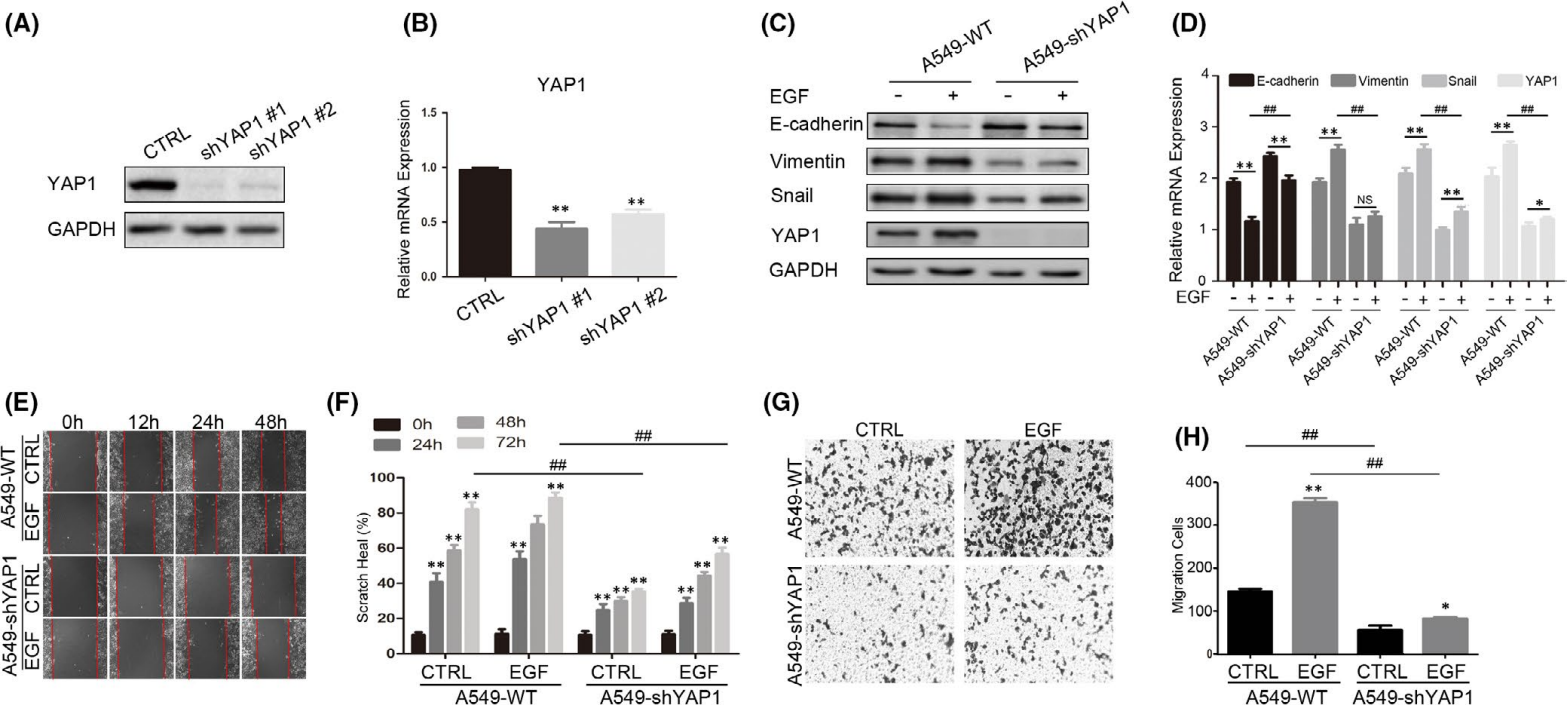
YAP1 knocking down impairs EGF‐induced epithelial‐mesenchymal transition (EMT). (A) Western Blots and qPCR (B) were carried to detect the knocking down efficiency of YAP1. (C,D) Cells were treated with 25 ng/ml EGF for 72 h, Western blot (C) and qPCR (D) were performed to detect the EMT‐related markers in A549‐WT and A549‐shYAP1. (E,G) Cells were pre‐treated with 25 ng/ml EGF for 72 h, the scratch healing assay (E) and trans‐well assay (G) were performed to detect the migration ability. (F,H) Statistic analysis of the scratch healing assay (F) and trans‐well assay (H). *p < 0.05, **p < 0.001 compared to control and ^##^ *p* < 0.001 compared to the linked group

To assess the potential role of YAP1 in the EMT process, endogenous YAP1 was suppressed in A549 cells using pLentiviral shRNA vector (Figure 3A,B). EMT markers were examined in A549‐shYAP1 cells and H460‐shYAP1 cells. Western blot analysis showed that the epithelial marker E‐cadherin was increased in the YAP1 knockdown cells, while the mesenchymal markers Vimentin and Snail were decreased, and these changes became more significant after EGF stimulation (Figure 3C and Figure S1A). The qPCR results show that the changes of mRNA and protein were basically consistent (Figure 3D and Figure S1B). Together, these results suggest that suppression of YAP1 expression could partially block the EGF‐induced EMT process in A549 and H460 cells. Cell scratch and trans‐well assays were then performed to determine the migration abilities of YAP1 knockdown cells. The results showed that the efficiency of wound healing (Figure 3E,F and Figure S1C,D) and the number of migrated cells (Figure 3G,H and Figure S1E,F) were significantly lower in YAP1 knockdown cells compared to the control either with or without EGF stimulation.

### 3.4 YAP1‐2 is the dominant isoform in the EMT process in A549 cells

**FIGURE 4:**
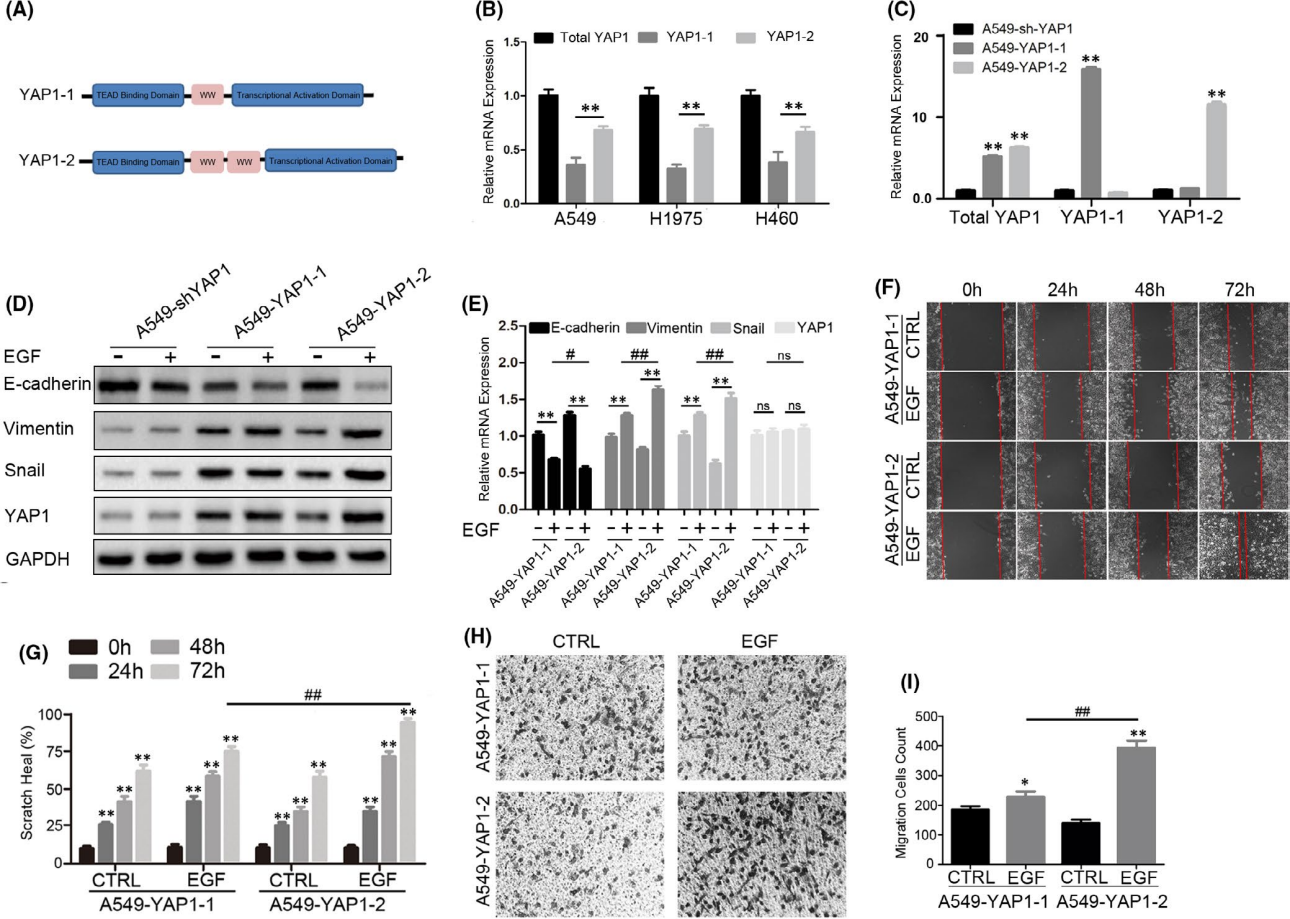
YAP1‐2 is an important factor in EGF‐induced A549 cells epithelial‐mesenchymal transition (EMT). (A) Schematic illustration of the structure of YAP1‐1 and YAP1‐2. (B) qPCR detection of the expression of total YAP1, YAP1‐1 and YAP1‐2. (C) qPCR analysis on the expression of total YAP1, YAP1‐1 and YAP1‐2 in A549‐shYAP1, A549‐YAP1‐1 and A549‐YAP1‐2. (D,E) Cells were treated with 25ng/ml EGF for 72 h, Western blots (D) and qPCR (E) were performed to detect the EMT‐related markers in A549‐shYAP1, A549‐YAP1‐1 and A549‐YAP1‐2. (F,H) Cells were pre‐treated with 25 ng/ml EGF for 72 h, the scratch healing assay (F) and trans‐well assay (H) were performed to detect the migration ability of A549‐YAP1‐1 and A549‐YAP1‐2. (G,I) Statistic analysis of the scratch healing assay (I) and trans‐well assay(K). *p < 0.05, **p < 0.001 compared to control and ^#^ *p* < 0.05, ^##^ *p* < 0.001 compared to the linked group

**FIGURE 5:**
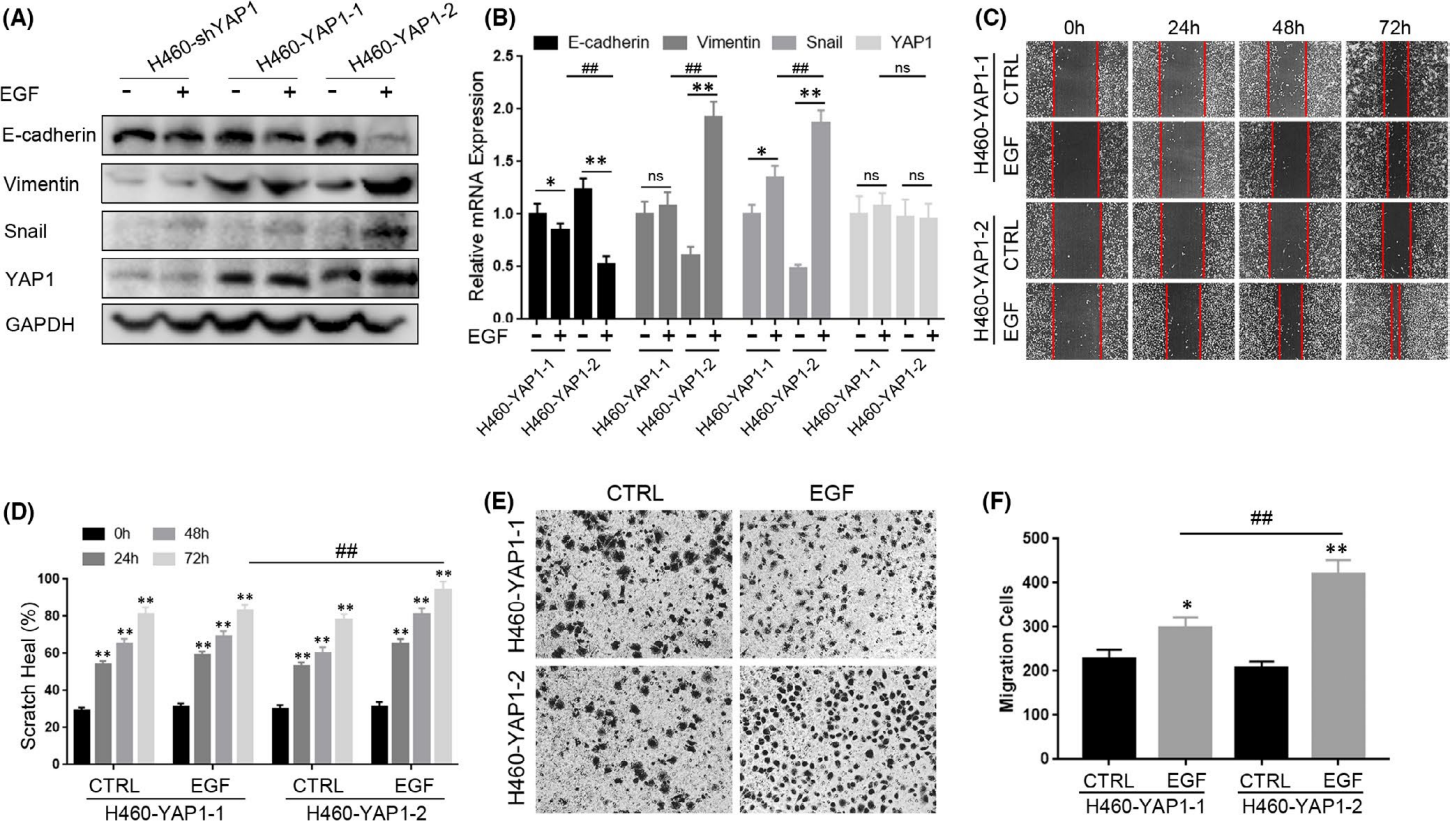
YAP1‐2 is an important factor in EGF‐induced lung cancer epithelial‐mesenchymal transition (EMT) in H460. (A,B) Cells were treated with 25 ng/ml EGF for 72 h, Western blots (A) and qPCR (B) were performed to detect the EMT‐related markers in H460‐shYAP1, H460‐YAP1‐1 and H460‐YAP1‐2. (C,E) Cells were pre‐treated with 25 ng/ml EGF for 72 h, the scratch healing assay (C) and trans‐well assay (E) were performed to detect the migration ability of H460‐YAP1‐1 and H460‐YAP1‐2. (D,F) Statistic analysis of the scratch healing assay (D) and trans‐well assay (F). *p < 0.05, **p < 0.001 compared to control and ^##^ *p* < 0.001 compared to the linked group

YAP1 isoforms can be divided into two subgroups according to their WW domains (Figure 4A). Specific primers targeting YAP1‐1 or YAP1‐2 were designed to detect the mRNA level of each isoform in NSCLC cells. The mRNA level of YAP1‐2 was much higher than that of YAP1‐1 in NSCLC cells (Figure 4B). To further investigate the difference between these two isoforms, A549 stable cell lines expressing a single YAP1 isoform were constructed based on A549‐shYAP1 (shYAP1 #1, the target is YAP1 3’ UTR sequence); these cells were named A549‐YAP1‐1 cells and A549‐YAP1‐2 cells, respectively. Western blots and qPCR confirmed efficient and specific overexpression of YAP1‐1 and YAP1‐2 isoforms in A549‐shYAP1 stable cells, respectively, at both the mRNA and the protein levels (Figure 4C,D). The isoform‐distinguishing qPCR results confirmed YAP1‐1 as the predominant isoform in A549‐YAP1‐1 cells and YAP1‐2 as the major isoform in A549‐YAP1‐2 cells, respectively (Figure 4C), therefore validated their expression efficiency and specificity. We also generated reconstitutively expressed YAP1 isoform in H460‐shYAP1 cells (Figure 5A). The expression of EMT markers was further evaluated in the A549‐YAP1‐1 and A549‐YAP1‐2 cells with and without EGF stimulation. The level of the epithelial marker E‐cadherin in the A549‐YAP1‐1 cells was lower than that in the A549‐YAP1‐2 cells, while the levels of the mesenchymal markers Vimentin and Snail were higher in the A549‐YAP1‐1 cells than in the A549‐YAP1‐2 cells. However, the relative amount of these markers changed in completely opposite ways when the cells were treated with EGF; lower expression of the E‐cadherin and higher expression of the mesenchymal markers were observed in the A549‐YAP1‐2 cells compared to the A549‐YAP1‐1 cells (Figure 4D,E). Similar results were obtained in H460 cells (Figure 5A,B). The results of scratch healing and trans‐well assays were consistent with the EMT marker detection. The efficiency of wound healing (Figure 4F,G) and the number of invading cells (Figure 4H,I) was higher in the A549‐YAP1‐1 cells than in the A549‐YAP1‐2 cells without EGF treatment. However, contrasting results were observed when the cells were treated with EGF. A similar phenomenon was also observed in H460 cells (Figure 5C‐F) suggesting that this isoform‐dependent regulation of EMT is a conserved among different cell NSCLC cells lines.

### 3.5 EGF promotes YAP1 protein stability and its nuclear localization

**FIGURE 6:**
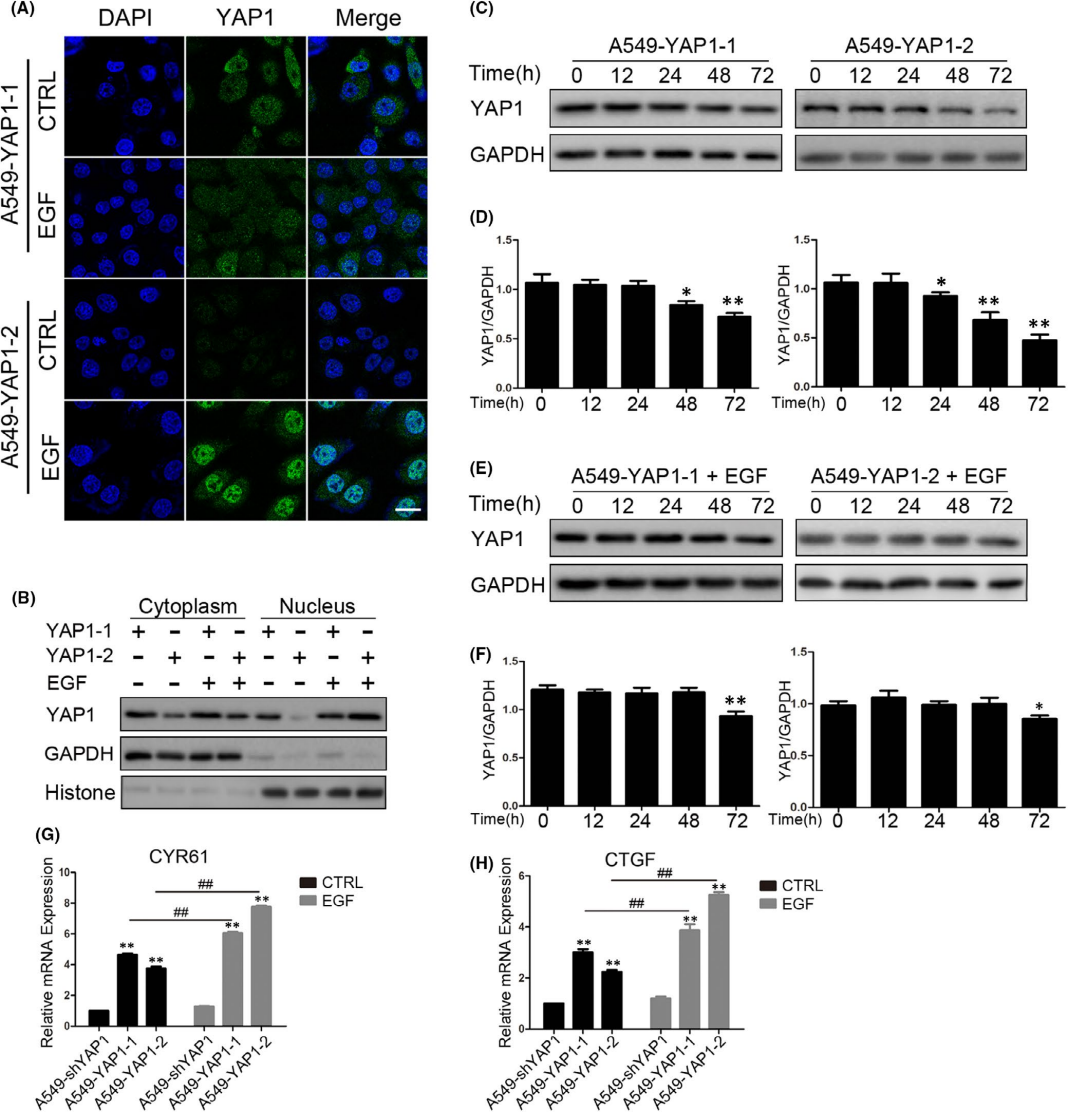
EGF accelerates YAP1‐2 nucleus localization and stability. (A) Cells were pre‐treated with 25 ng/ml EGF for 72 h, immunofluorescence was performed to detect the expression and cellular localization of YAP1 in A549‐YAP1‐1 and A549‐YAP1‐2. (B) Cells were pre‐treated with 25 ng/ml EGF for 72 h, cytoplasmic nucleus protein isolation was then performed to detect the distribution of YAP1 in A549‐YAP1‐1 and A549‐YAP1‐2 cells. (C) A549‐YAP1‐1 and A549‐YAP1‐2 cells were cultured in low cell density condition for 3 days to stabilize YAP1 expression with or without EGF (25 ng/ml) treatment. The cells were then transferred to 35 mm dishes with high cell density to trigger protein degradation. Whole cell lysates of A549‐YAP1‐1 and A549‐YAP1‐2 cells were collected at indicated time points and subjected to Western blots to detect the abundance of YAP1. (D) Statistic analysis of (C). (E) Cells were pre‐treated with 25 ng/ml EGF for 72 h in low cell density and then translocated to high cell density to detect the degradation of YAP1 in A549‐YAP1‐1 and A549‐YAP1‐2 cells. (F) Statistic analysis of E. (G,H) Cells were pre‐treated with 25 ng/ml EGF for 72 h, and qPCR was performed to detect the mRNA level of YAP1‐downstream target CYR61 (G) and CTGF (H) in A549‐ShYAP1, A549‐YAP1‐1 and A549‐YAP1‐2 cells. *p < 0.05, **p < 0.001 compared to control and ^##^ *p* < 0.001 compared to the linked group

As YAP1 nuclear localization is vital for its regulation and activity, we next examined the subcellular localization of YAP1 in A549‐YAP1‐1 and A549‐YAP1‐2 cells with and without EGF stimulation using immunofluorescence. In non‐treated cells, more nuclear‐localized YAP1 in the A549‐YAP1‐1 cells than in the A549‐YAP1‐2 cells were observed. However, upon treatment with EGF, the overall protein level of YAP1 in the A549‐YAP1‐2 cells was significantly increased accompanying with more robust nuclear translocation, while no significant changes were observed in the A549‐YAP1‐1 cells (Figure 6A). Western blot analyses on the cytoplasmic and nuclear‐fractionated protein samples were consistent with the immunofluorescence. We observed higher expression of YAP1 in the cytoplasm and nucleus in A549‐YAP1‐1 cells than in A549‐YAP1‐2 cells in control group. Significantly, upon treatment with EGF, total YAP1 were increased in both cell lines, in particular, nuclear YAP1 was even higher in A549‐YAP1‐2 cells, than that in A549‐YAP1‐1 cells (Figure 6B). The degradation of YAP1‐2 cells was stronger than YAP1‐1 under normal culture condition (Figure 6C,D); however, this trend was reversed after EGF stimulation (Figure 6E,F). We determined the mRNA expression of CYR61 and CTGF, two downstream target genes of YAP1, to assess the transcriptional activity of YAP1. Without EGF stimulation, the expression of CYR61 and CTGF in the A549‐YAP1‐1 cells was higher than in the A549‐YAP1‐2 cells. However, when stimulated with EGF, the expression of CYR61 and CTGF in the A549‐YAP1‐2 cells became higher than those in the A549‐YAP1‐1 cells (Figure 6G,H).

### 3.6 EGF enhances YAP1 protein stability via the AKT pathway

**FIGURE 7:**
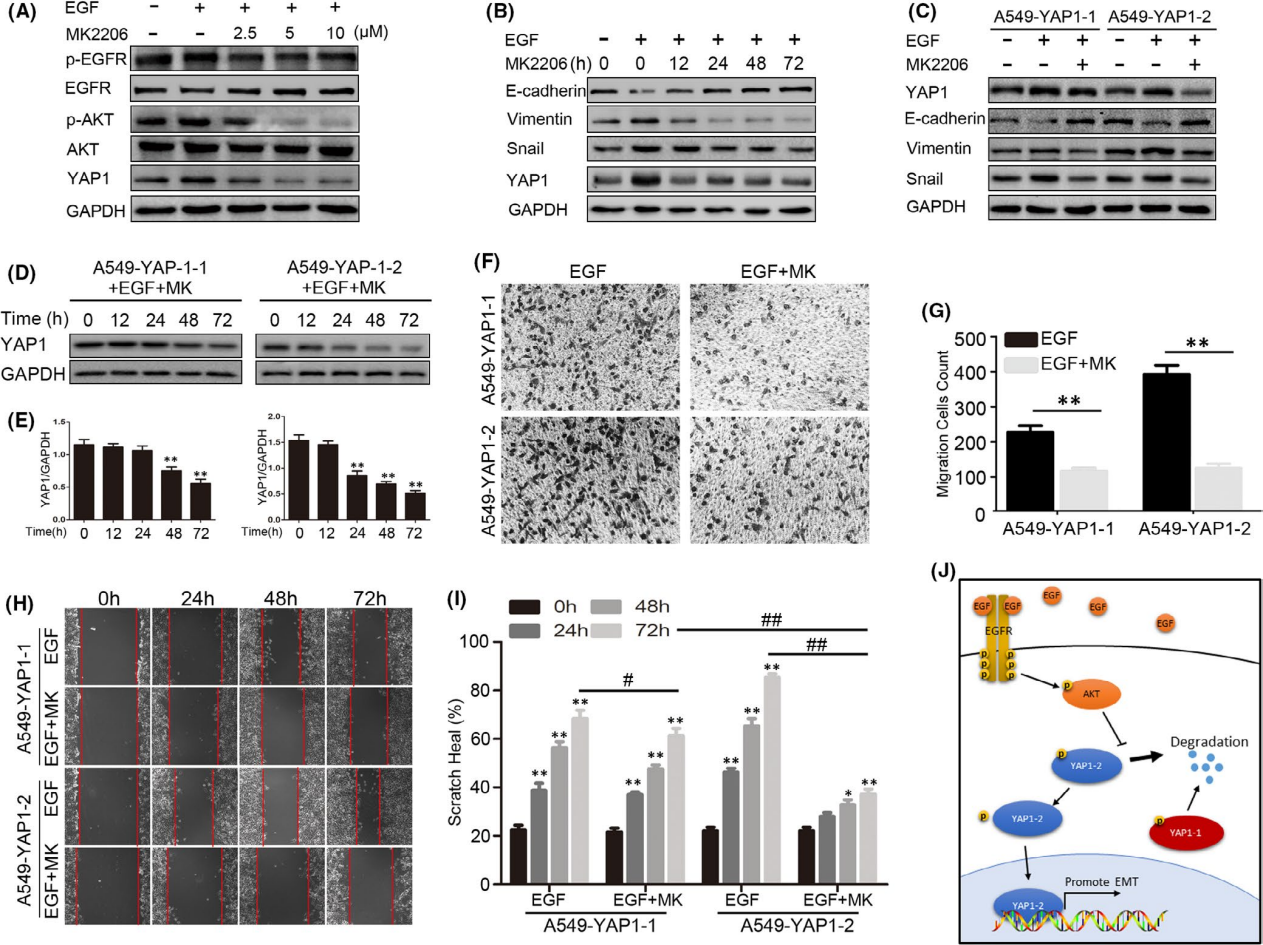
EGF promotes YAP1 by activating AKT. (A) Western blots were used to analyse the inhibition efficiency of MK2206 in A549 on AKT pathway through a dose‐dependent manner. (B) Cells were pre‐treated with 25 ng/ml EGF for 72 h before MK2206 (2.5 μM) treatment and cells were collected at different time point, Western blot was performed to detect the EMT‐related markers in A549. (C) Cells were pre‐treated with 25 ng/ml EGF for 72 h before MK2206(2.5 μM) treatment, Western blot was performed to detect the EMT‐related markers in A549‐YAP1‐1 and A549‐YAP1‐2 after 24 h. (D) Cells were pre‐treated with 25 ng/ml EGF and 2.5 μM MK2206 for 72 h in low cell density and then translocated to high cell density to detect the degradation of YAP1 in A549‐YAP1‐1 and A549‐YAP1‐2 cells. (E) Statistic analysis of D. (F,H) Cells were pre‐treated with 25 ng/ml EGF and 2.5 μM MK2206 for 72 h, the trans‐well assay (F) and scratch healing assay (H) were performed to detect the migration ability of A549‐YAP1‐1 and A549‐YAP1‐2. (G,I) Statistic analysis of the trans‐well assay (G) and scratch healing assay (I). *p < 0.05, **p < 0.001 compared to control and ^#^ *p* < 0.05, ^##^ *p* < 0.001 compared to the linked group. (J) The regulatory network of YAP1‐1 and YAP1‐2 in EGF‐induced EMT progression

EGF and its receptors are widely distributed in different cell types and can activate multiple downstream pathways. It has been reported that the AKT signalling pathway regulates the protein stability of YAP1.^21^ Here, we applied AKT‐specific inhibitor MK2206 to investigate the role of this pathway in YAP1‐mediated EMT. As expected, a dose‐dependent inhibitory effect of MK2206 on EGF activation of phospho‐AKT was observed at 2.5 μM and above in A549 cells, which correlated to a decrease in the level of YAP1 protein (Figure 7A). Time kinetics analysis further indicated that treatment with MK2206 partially rescued EGF‐induced EMT and YAP1 upregulation (Figure 7B). Based on the above observation, we treated A549‐YAP1‐1 and A549‐YAP1‐2 cells with 5 μM of MK2206 for 24 h, then, the protein samples were harvested for expression analysis of EMT markers. As shown in Figure 7C, EGF‐induced EMT phenotype was inhibited by MK2206, and the effect on A549‐YAP1‐2 cells was much stronger than that on A549‐YAP1‐1 cells. Interestingly, MK2206 treatment led to stronger degradation of YAP1‐2 compared to YAP1‐1 (Figure 7D,E) suggesting that AKT inhibition impairs EGF‐induced EMT, and the protein stability of YAP1‐2 is more sensitive to AKT inhibition. The effect of MK2206 on the invasion and metastasis in A549‐YAP1‐1 and A549‐YAP1‐2 cells was then determined using cell scratch and trans‐well assays. A stronger inhibition of MK2206 on the invasion and metastasis in A549‐YAP1‐2 cells was observed than in A549‐YAP1‐1 cells (Figure 7F‐I). Based on our research, we drew a cartoon to illustrate the regulatory network of YAP1‐1 and YAP1‐2 in EGF‐induced EMT progression (Figure 7J).

## 4 DISCUSSION

We recently reported the first systemic characterization and comparison of YAP1 isoforms with regard to their mRNA/protein expression, nuclear localization, transcription activation and functional properties.^10^ Consistent with the dissociated expression between the mRNA and protein of YAP1‐1 and YAP‐2 isoforms in pancreatic cancer cells, we observed lower expression of YAP1‐2 protein in NSCLC, despite its higher expression at mRNA level compared to YAP1‐1. This is likely due to stronger interactions of YAP1‐2 with some of negative regulators such as LATS1/2 as we previously demonstrated in the setting of high cell density,^10^ but the biological and functional differences between the YAP1 isoforms, particularly their potential distinctive response to various regulatory signalling remain largely unexplored.

The aberrantly activated EGFR pathway by gene amplification and/or mutation is highly frequent in NSCLC, particularly in patients of East Asian descent.^22^ EGFR targeting inhibitors have been widely used in clinical treatment,^23^ yet the regulatory machinery underlying its oncogenic activity is still not fully understood. The current work illustrated important roles of YAP1 isoforms in EGF‐induced EMT progression in NSCLC. Interestingly, YAP1 isoforms exhibited different responses to EGF stimulation, in that the protein stability of YAP1‐2 and its nuclear localization was enhanced more compared to YAP1‐1. Moreover, our recent findings suggest that a similar phenomenon exists in TGF‐β induced EMT in pancreatic cancer.^24^ The identification of factors that influence the stability of YAP1 isoforms may lead to the discovery of possible determinants of NSCLC progression. In this regard, we postulate that the extra WW domain of YAP1‐2 may contribute to the higher overall stability of the nuclear‐localized YAP1‐2 through binding particular molecules that may also modulate its transcriptional activity, when compared to YAP1‐1.

Although YAP1‐2 exhibited stronger response to EGF stimulation, the overall amount of YAP1‐2 localized to the nuclei was comparable to that of YAP1‐1 due to a higher basal level of YAP1‐1. Therefore, mere nuclear localization cannot fully explain the more robust effect of YAP1‐2 in promoting invasion and metastasis of cancer cells than YAP1‐1 upon EGF treatment. We suggest that the enhanced activity of YAP1‐2 on EMT may also involve stronger impact of EGF on the transcriptional activity of YAP1‐2 than YAP1‐1 on the downstream target genes. The presence of the extra WW domain may confer additional protein binding specificity/affinity enabling YAP1‐2 to selectively bind distinct/particular nuclear proteins that may be critical for various cellular activities, including promotion of cell malignancy. The WW domain not only binds to negative regulators but also interacts with nuclear factors, such as ZEB1, RUNX, P73, Slug and SMADs, and alters the targets of YAP1.^25^, ^26^, ^27^, ^28^ Among these factors, ZEB1 and SMADs are important EMT‐regulating factors, whose expression are already elevated. Various mechanisms have been reported for the YAP1 to promote EMT including two recent studies indicating YAP1 and ZEB1 complex formation to activate ITGA3 transcription, which is marker and a driver of EMT,^18^ its high expression has recently been found to correlate with poor prognosis in NSCLC patients.^29^ However, none of the studies have taken into consideration of YAP1 isoforms and their potential differential impact on this process.

Based on the current result and previous reports, we can speculate that during the early stage of cancer, when cancer cells exhibit low malignancy, YAP1‐1 binds weakly to negative regulators and shows higher protein stability and nuclear localization than YAP1‐2. However, this situation changes when cancer cells undergo EMT and malignant transformation. With enhanced stability and nuclear translocation, YAP1‐2 is more potent in promoting tumour invasion and metastasis than YAP1‐1. Therefore, we presume that YAP1‐1 might act as the dominant isoform in tumours with low malignancy, while YAP1‐2 might act more important in tumours with high malignancy. Although the precise mechanism underlying the superiority of YAP1‐2 over YAP1‐1 in promoting tumour invasion and metastasis remains to be determined, one plausible explanation is that the extra WW domain of YAP1‐2 allows its binding of additional nuclear partners, some of which may be critical drivers of EMT. Therefore, when we wish to target YAP1 for cancer treatment, we should at least clarify which isoform of YAP1 is the dominant one before treatment strategies are undertaken. Furthermore, the finding that Yap1 isoforms are differentially expressed and regulated in NSCLC may lead to identification of novel Yap1‐1 or Yap1‐2 isoform‐based biomarkers with diagnostic value. In addition, other aspects of Yap1 isoforms such as possession of distinct WW domains between them could also be therapeutically exploited and potentially prove beneficial for the treatment of NSCLC as well as other cancers.

Our data also indicate that the AKT pathway is critical for YAP1‐2 stability, which is consistent with previous reports.^30^ Although Hippo pathway is the main upstream regulator of YAP1, mainly by cell contacts, the AKT pathway may become important for cancer cells undergoing EMT. During EMT process, the cell contacts become weaker and Hippo signalling is inhibited, leading to YAP1‐2 stabilization and enhanced nuclear localization. Therefore, AKT signalling may promote YAP1‐2 stabilization and nuclear localization in both direct and indirect ways. Future studies will investigate the possibility that EGF signalling may differentially impact the interactions of nuclear YAP1 isoforms with EMT‐related nuclear factors during the EMT process and their localization as well as their transcriptional activities.

## CONFLICT OF INTERESTS

The authors declare that the research was conducted in the absence of any commercial or financial relationships that could be construed as a potential conflict of interest.

## AUTHOR CONTRIBUTION

**Qiang Guo:** Formal analysis (lead); Methodology (equal); Project administration (equal); Writing – original draft (equal). **Mei‐Yu Quan:** Methodology (equal); Project administration (equal); Writing – original draft (equal). **Le Xu:** Methodology (supporting). **Yaxin Cai:** Methodology (supporting). **Jue‐Ting Cai:** Methodology (supporting). **Xue Li:** Methodology (supporting). **Guifeng Feng:** Methodology (supporting). **Aiping Chen:** Methodology (supporting). **Weiwei Yang:** Methodology (supporting). **Qhaweni Dhlamini:** Writing – original draft (supporting). **Tian‐Fang Jiang:** Methodology (supporting). **Chengguo Shen:** Formal analysis (supporting). **Chengshui Chen:** Conceptualization (equal); Funding acquisition (supporting); Project administration (equal). **Jin‐San Zhang:** Conceptualization (equal); Funding acquisition (lead); Supervision (equal); Writing – review & editing (lead).

## Supporting information

Figure S1

## Data Availability

The data sets used and analyzed during the current study are available from the corresponding author on reasonable request.

## References

[1] Harrison P , Vyse S , Huang P . Rare epidermal growth factor receptor (EGFR) mutations in non‐small cell lung cancer. Semin Cancer Biol. 2020;61:167‐179. doi:10.1016/j.semcancer.2019.09.015 31562956 PMC7083237

[2] Fois S , Paliogiannis P , Zinellu A , Fois A , Cossu A , Palmieri G . Molecular epidemiology of the main druggable genetic alterations in non‐small cell. Lung Cancer. 2021;22(2):612. doi:10.3390/ijms22020612 PMC7827915 33435440

[3] Siegel RL , Miller KD , Jemal A . Cancer statistics, 2020. CA Cancer J Clin. 2020;70(1):7‐30. doi:10.3322/caac.21590 31912902

[4] Dongre A , Weinberg RA . New insights into the mechanisms of epithelial‐mesenchymal transition and implications for cancer. Nat Rev Mol Cell Bio. 2019;20(2):69‐84. doi:10.1038/s41580-018-0080-4 30459476

[5] Brabletz T , Kalluri R , Nieto MA , Weinberg RA . EMT in cancer. Nat Rev Cancer. 2018;18(2):128‐134. doi:10.1038/nrc.2017.118 29326430

[6] Udan RS , Kango‐Singh M , Nolo R , Tao C , Halder G . Hippo promotes proliferation arrest and apoptosis in the Salvador/Warts pathway. Nat Cell Biol. 2003;5(10):914‐920. doi:10.1038/ncb1050 14502294

[7] Meng Z , Moroishi T , Guan KL . Mechanisms of Hippo pathway regulation. Genes Dev. 2016;30(1):1‐17. doi:10.1101/gad.274027.115 26728553 PMC4701972

[8] Gregorieff A , Wrana JL . Hippo signalling in intestinal regeneration and cancer. Curr Opin Cell Biol. 2017;48:17‐25. doi:10.1016/j.ceb.2017.04.005 28527754

[9] Levy D , Adamovich Y , Reuven N , Shaul Y . Yap1 phosphorylation by c‐Abl is a critical step in selective activation of proapoptotic genes in response to DNA damage. Mol Cell. 2008;29(3):350‐361. doi:10.1016/j.molcel.2007.12.022 18280240

[10] Guo Q , Quan M , Dong J , et al. The WW domains dictate isoform‐specific regulation of YAP1 stability and pancreatic cancer cell malignancy. Theranostics. 2020;10(10):4422‐4436. doi:10.7150/thno.42795 32292505 PMC7150473

[11] Sudol M . YAP1 oncogene and its eight isoforms. Oncogene. 2013;32(33):3922. doi:10.1038/onc.2012.520 23160371

[12] Sudol M , Harvey KF . Modularity in the Hippo signaling pathway. Trends Biochem Sci. 2010;35(11):627‐633. doi:10.1016/j.tibs.2010.05.010 20598891

[13] Pan D . The hippo signaling pathway in development and cancer. Dev Cell. 2010;19(4):491‐505. doi:10.1016/j.devcel.2010.09.011 20951342 PMC3124840

[14] Leung CY , Zernicka‐Goetz M . Angiomotin prevents pluripotent lineage differentiation in mouse embryos via Hippo pathway‐dependent and ‐independent mechanisms. Nat Commun. 2013;4:2251. doi:10.1038/ncomms3251 23903990 PMC3741640

[15] McDonald CB , McIntosh SK , Mikles DC , et al. Biophysical analysis of binding of WW domains of the YAP2 transcriptional regulator to PPXY motifs within WBP1 and WBP2 adaptors. Biochemistry. 2011;50(44):9616‐9627. doi:10.1021/bi201286p 21981024 PMC3210484

[16] Liang G , Duan C , He J , Ma W , Dai X . PTPN14, a target gene of miR‐4295, restricts the growth and invasion of osteosarcoma cells through inactivation of YAP1 signalling. Clin Exp Pharmacol Physiol. 2020;47(7):1301‐1310. doi:10.1111/1440-1681.13296 32141101

[17] Iglesias‐Bexiga M , Castillo F , Cobos E , Oka T , Sudol M , Luque I . WW domains of the yes‐kinase‐associated‐protein (YAP) transcriptional regulator behave as independent units with different binding preferences for PPxY motif‐containing ligands. PLoS One. 2015;10(1):e0113828. doi:10.1371/journal.pone.0113828 25607641 PMC4301871

[18] Liu M , Zhang Y , Yang J , et al. Zinc dependent regulation of ZEB1 and YAP1 co‐activation promotes EMT plasticity and metastasis in pancreatic cancer. Gastroenterology. 2021;160(5):1771‐1783. doi:10.1053/j.gastro.2020.12.077 33421513 PMC8035249

[19] Ji L , Li X , Zhou Z , Zheng Z , Jin L , Jiang F . LINC01413/hnRNP‐K/ZEB1 axis accelerates cell proliferation and EMT in colorectal cancer via inducing YAP1/TAZ1 translocation. Mol Ther Nucleic Acids. 2020;19:546‐561. doi:10.1016/j.omtn.2019.11.027 31927328 PMC6953771

[20] Zhang JS , Herreros‐Villanueva M , Koenig A , et al. Differential activity of GSK‐3 isoforms regulates NF‐kappaB and TRAIL‐ or TNFalpha induced apoptosis in pancreatic cancer cells. Cell Death Dis. 2014;5:e1142. doi:10.1038/cddis.2014.102 24675460 PMC4067531

[21] Hu LL , Su T , Luo RC , et al. Hippo pathway functions as a downstream effector of AKT signaling to regulate the activation of primordial follicles in mice. J Cell Physiol. 2019;234(2):1578‐1587. doi:10.1002/jcp.27024 30078193

[22] Rosell R , Bivona TG , Karachaliou N . Genetics and biomarkers in personalisation of lung cancer treatment. Lancet. 2013;382(9893):720‐731. doi:10.1016/S0140-6736(13)61715-8 23972815

[23] Jiang J , Huang L , Liang X , et al. Gefitinib versus docetaxel in previously treated advanced non‐small‐cell lung cancer: a meta‐analysis of randomized controlled trials. Acta Oncol. 2011;50(4):582‐588. doi:10.3109/0284186X.2010.546368 21190510

[24] Gao C , Quan M‐Y , Chen Q‐J , et al. Yap1‐2 isoform is the primary mediator in TGF‐β1 induced EMT in pancreatic cancer. Front Oncol. 2021;11:1‐2.. doi:10.3389/fonc.2021.649290 PMC8170464 34094936

[25] Lehmann W , Mossmann D , Kleemann J , et al. ZEB1 turns into a transcriptional activator by interacting with YAP1 in aggressive cancer types. Nat Commun. 2016;7:10498. doi:10.1038/ncomms10498 26876920 PMC4756710

[26] Yao M , Wang Y , Zhang P , et al. BMP2‐SMAD signaling represses the proliferation of embryonic neural stem cells through YAP. J Neurosci. 2014;34(36):12039‐12048. doi:10.1523/jneurosci.0486-14.2014 25186749 PMC6608470

[27] Levy D , Reuven N , Shaul Y . A regulatory circuit controlling Itch‐mediated p73 degradation by Runx. J Biol Chem. 2008;283(41):27462‐27468. doi:10.1074/jbc.M803941200 18701449

[28] Yu M , Chen Y , Li X , et al. YAP1 contributes to NSCLC invasion and migration by promoting Slug transcription via the transcription co‐factor TEAD. Cell Death Dis. 2018;9(5):464. doi:10.1038/s41419-018-0515-z 29700328 PMC5920099

[29] Shirakihara T , Kawasaki T , Fukagawa A , et al. Identification of integrin α3 as a molecular marker of cells undergoing epithelial‐mesenchymal transition and of cancer cells with aggressive phenotypes. Cancer Sci. 2013;104(9):1189‐1197. doi:10.1111/cas.12220 23786209 PMC7656537

[30] Borreguero‐Munoz N , Fletcher GC , Aguilar‐Aragon M , Elbediwy A , Vincent‐Mistiaen ZI , Thompson BJ . The Hippo pathway integrates PI3K‐Akt signals with mechanical and polarity cues to control tissue growth. PLoS Biol. 2019;17(10):e3000509. doi:10.1371/journal.pbio.3000509 31613895 PMC6814241

