# Supplementary material for: Enhanced nuclear localization of YAP1‐2 contributes to EGF‐induced EMT in NSCLC
Source: J Cell Mol Med. 2022 Jan 11;26(4):1013–23. doi: 10.1111/jcmm.17150 (PMC8831977; doi:10.1111/jcmm.17150)
Supplement: Supplementary file 1 — Figure S1 [file JCMM-26-1013-s001.docx]

**Supplementary Figure 1.**


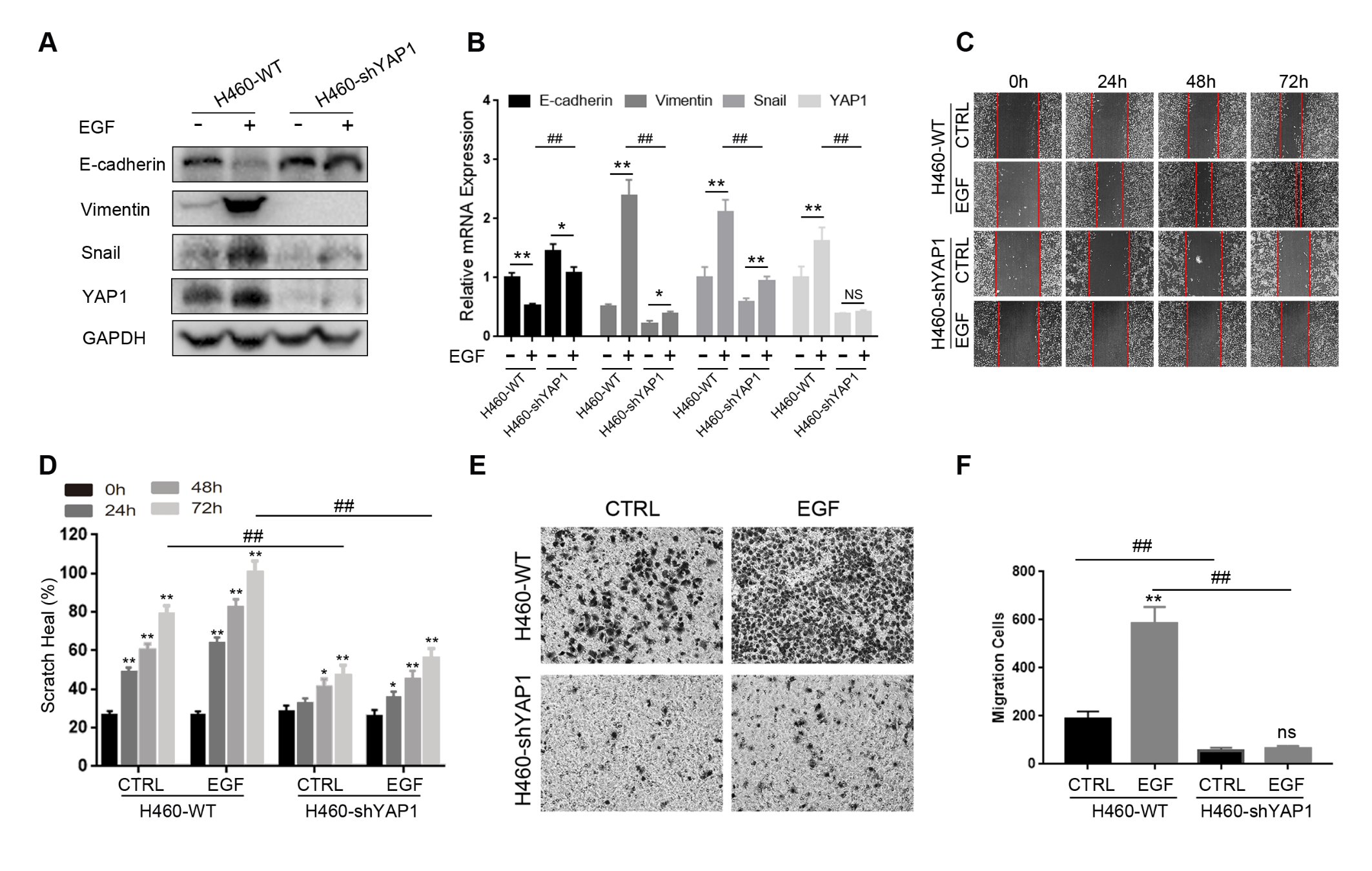


**YAP1 knocking down impairs EGF-induced EMT in H460 cells.**

(A, B) Cells were treated with 25ng/ml EGF for 72 h, Western Blot (A) and qPCR(B) were performed to detect the EMT related markers in H460-WT and H460-ShYAP1. (C, E) Cells were pretreated with 25ng/ml EGF for 72 h, the scratch healing assay(C) and trans-well assay(E) were performed to detect the migration ability. (D, F) Statistic analysis of the scratch healing assay (D) and trans-well assay(F). *p<0.05, **p<0.001 compared to control and #P<0.05，##P<0.001 compared to the linked group.
